# A *Rickettsiella* Endosymbiont Is a Potential Source of Essential B-Vitamins for the Poultry Red Mite, *Dermanyssus gallinae*

**DOI:** 10.3389/fmicb.2021.695346

**Published:** 2021-09-03

**Authors:** Daniel R. G. Price, Kathryn Bartley, Damer P. Blake, Eleanor Karp-Tatham, Francesca Nunn, Stewart T. G. Burgess, Alasdair J. Nisbet

**Affiliations:** ^1^Moredun Research Institute, Penicuik, United Kingdom; ^2^Department of Pathobiology and Population Sciences, Royal Veterinary College, London, United Kingdom

**Keywords:** endosymbiont, mutualist, Gammaproteobacteria, vitamin biosynthesis, hematophagous, microbiome

## Abstract

Many obligate blood-sucking arthropods rely on symbiotic bacteria to provision essential B vitamins that are either missing or at sub-optimal levels in their nutritionally challenging blood diet. The poultry red mite *Dermanyssus gallinae*, an obligate blood-feeding ectoparasite, is a serious threat to the hen egg industry. Poultry red mite infestation has a major impact on hen health and welfare and causes a significant reduction in both egg quality and production. Thus far, the identity and biological role of nutrient provisioning bacterial mutualists from *D. gallinae* are little understood. Here, we demonstrate that an obligate intracellular bacterium of the *Rickettsiella* genus is detected in *D. gallinae* mites collected from 63 sites (from 15 countries) across Europe. In addition, we report the genome sequence of *Rickettsiella* from *D. gallinae* (*Rickettsiella – D. gallinae* endosymbiont; *Rickettsiella* DGE). *Rickettsiella* DGE has a circular 1.89Mbp genome that encodes 1,973 proteins. Phylogenetic analysis confirms the placement of *Rickettsiella* DGE within the *Rickettsiella* genus, related to a facultative endosymbiont from the pea aphid and *Coxiella*-like endosymbionts (CLEs) from blood feeding ticks. Analysis of the *Rickettsiella* DGE genome reveals that many protein-coding sequences are either pseudogenized or lost, but *Rickettsiella* DGE has retained several B vitamin biosynthesis pathways, suggesting the importance of these pathways in evolution of a nutritional symbiosis with *D. gallinae*. *In silico* metabolic pathway reconstruction revealed that *Rickettsiella* DGE is unable to synthesize protein amino acids and, therefore, amino acids are potentially provisioned by the host. In contrast, *Rickettsiella* DGE retains biosynthetic pathways for B vitamins: thiamine (vitamin B1) *via* the salvage pathway; riboflavin (vitamin B2) and pyridoxine (vitamin B6) and the cofactors: flavin adenine dinucleotide (FAD) and coenzyme A (CoA) that likely provision these nutrients to the host.

## Introduction

Animals live in a diverse bacterial world and mutualistic associations with bacteria can provide these animals with novel biochemical traits to exploit an otherwise inaccessible ecological niche (McFall-Ngai et al., 2013). For example, specialist phloem-feeding insects of the order Hemiptera depend on bacterial endosymbionts to synthesize and provide essential amino acids that are largely absent in their phloem sap diet (Moran, 2007). Similarly, obligate blood-feeding arthropods, including insects, ticks, and mites associate with nutritional mutualists that provide essential vitamins and cofactors that are in limited supply from their blood diet (recently reviewed in Husnik, 2018). Typically, as a result of relaxed selection, the genomes of nutritional mutualists are reduced relative to their free-living ancestors. Genes that are essential for symbiosis are retained, while non-essential genes are lost, resulting in small compact genomes (as reviewed by Toft and Andersson, 2010; McCutcheon and Moran, 2012). The microbiome of obligate blood-feeding invertebrates is often dominated by a single B vitamin provisioning symbiont. For example, the blood-feeding African soft tick (*Ornithodoros moubata*) is associated with a *Francisella* (strain F-Om) that provides the host with essential B vitamins to supplement its blood meal diet (Duron et al., 2018). The genome sequence of *Francisella* F-Om bears the hallmarks of a host-adapted bacterial endosymbiont, with dramatic genome reduction resulting from loss of redundant genes that are not required for a symbiotic function. Importantly, *Francisella* F-Om retains biosynthesis pathways for B vitamins biotin (B7), riboflavin (B2), folic acid (B9) and cofactors coenzyme A (CoA) and flavin adenine dinucleotide (FAD) to supplement deficiencies in the hosts diet (Duron et al., 2018). This pattern of genome reduction and retention of B vitamin biosynthesis pathways is also observed in *Coxiella*-like endosymbionts (CLEs) from obligate blood-feeding ticks. Recent genome sequence studies revealed that, in comparison to the pathogen *Coxiella burnetii* (genome size 2.03Mbp), CLEs from ticks have reduced genomes, as small as 0.66Mbp for CLE from the lone star tick (CLE of *Amblyomma americanum*). Yet, these nutritional mutualists retain pathways for B vitamin and cofactor biosynthesis to supplement the nutritional requirements of their blood-feeding host (Smith et al., 2015).

The poultry red mite (*Dermanyssus gallinae*) is an obligate blood-feeding ectoparasite that feeds on avian blood. This mite has a worldwide distribution and is endemic in many commercial poultry farms, with up to 83% of European egg-laying facilities infested by *D. gallinae* (George et al., 2015). Infestation of poultry houses has a serious impact on hen health and welfare and causes a significant reduction in both egg quality and production. Infestations can reach up to 500,000 mites per bird and cause welfare issues, including anemia, irritation, and even death of hens by exsanguination (Sigognault Flochlay et al., 2017). In the EU, *D. gallinae* infestation costs the poultry industry in excess of €231million *per annum* due to production losses alone (Sigognault Flochlay et al., 2017). In addition, *D. gallinae* has been implicated in the transmission of avian viral and bacterial disease (Huong et al., 2014; Sigognault Flochlay et al., 2017).

To utilize blood as a food source, our current hypothesis is that *D. gallinae* associates with bacterial mutualists, which synthesize and supply essential B vitamins and cofactors that are absent in the blood diet of the mite. A previous microbiome analysis of *D. gallinae* demonstrates that adult female mites have a simple microbiome, with 10 operational taxonomic units (OTUs) accounting for between 90 and 99% of the observed microbial diversity (Hubert et al., 2017). Furthermore, only four bacterial taxa, including: *Bartonella*, *Cardinium*, *Wolbachia*, and *Rickettsiella*, were present across all *D. gallinae* life-stages (Hubert et al., 2017). Data presented here, based on bacterial 16S rRNA amplicon sequencing confirms the presence of *Rickettsiella* in *D. gallinae* eggs, in agreement with previous studies (De Luna et al., 2009; Hubert et al., 2017). Here, we investigate the distribution of the previously identified *Rickettsiella – D. gallinae* endosymbiont (*Rickettsiella* DGE; Hubert et al., 2017) in *D. gallinae* across Europe, determine the complete genome of *Rickettsiella* DGE and examine this genome for evidence of biosynthesis pathways, which would supplement the diet of its host, *D. gallinae*.

## Materials and Methods

### Mite Collection and Endosymbiont-Enriched DNA Preparation

*Dermanyssus gallinae* were collected from a single commercial laying hen facility in the Scottish Borders, United Kingdom and maintained in 75cm^2^ canted tissue culture flasks (Corning Inc., Corning, NY, United States) at 4°C for up to 4weeks after collection. For experiments requiring mite eggs, freshly collected mixed stage and gender mites were placed into vented 25ml Sterilin universal tubes and maintained at 25°C, 75% relative humidity in a Sanyo MLR-350H incubator and eggs were collected the following day.

Since obligate bacterial endosymbionts are uncultivable outside the host, bacteria were derived from *D. gallinae* tissue lysates and host cells were removed from the extract using host depletion solution (Zymo Research, Irvine, CA, United States). Briefly, live mixed life-stage mites were surface sterilized with 70% (*v*/*v*) ethanol for 30s at room temperature followed by three 1min washes in nuclease-free water. Mites (approx. 25mg) were then homogenized in 200μl nuclease-free water using a tube pestle and host cells lysed by addition of 1ml of host depletion solution (Zymo Research, Irvine, CA, United States) with a 15min incubation at room temperature with end over end mixing. Intact bacterial cells were pelleted by centrifugation at 10,000×*g* for 5min at room temperature and DNA extracted from the pellet using a DNeasy® Blood & Tissue kit (Qiagen, Hilden, Germany). DNA concentration was assessed by the Qubit™ dsDNA BR Assay Kit (Thermo Fisher Scientific, Waltham, MA, United States) and 1% (*w*/*v*) agarose/TAE gel electrophoresis.

### 16S rRNA Amplicon Sequencing and Classification

Poultry red mite eggs were collected as described above using mites from the same commercial laying hen facility as described in section “Mite Collection and Endosymbiont-Enriched DNA Preparation.” Mite eggs were surface sterilized by two 5min washes in 0.1% (*w*/*v*) benzalkonium chloride followed by two 5min washes in 70% (*v*/*v*) ethanol. DNA was extracted from eggs using a DNeasy® Blood & Tissue kit (Qiagen, Hilden, Germany) with a lysozyme pre-treatment to lyse bacterial cells. DNA was quantified using a NanoDrop™ One spectrophotometer (Thermo Fisher Scientific, Waltham, MA, United States) and DNA molecular weight determined on a 1% (*w*/*v*) agarose/TAE gel. A reagent-only control DNA extraction was performed in parallel using the same DNA extraction kit.

The presence of bacterial DNA in mite eggs was verified by PCR using universal bacterial 16S rRNA gene primers 27F-short (5'-GAGTTTGATCCTGGCTCA-3') and 1507R (5'-TACCTTGTTACGACTTCACCCCAG-3'). Each 50μl PCR reaction contained template DNA (100ng), 1U Platinum™ *Taq* DNA Polymerase (Thermo Fisher Scientific, Waltham, MA, United States), 1× PCR buffer, 1.5mM MgCl_2_, 0.2mM of each dNTP and each primer at 0.2μM. Cycling conditions were as follows: 94°C for 2min; 30 cycles of 94°C 30s, 58°C 30s, 72°C 1min 30s, and a final hold of 72°C for 10min. A control PCR reaction was performed using the same conditions with an equivalent volume of eluate from the reagent-only control extraction. PCR products were cloned into pJET1.2 using the CloneJet PCR cloning kit (Thermo Fisher Scientific, Waltham, MA, United States) and transformed into chemically competent JM109 *Escherichia coli* cells (Promega, Madison, WI, United States). Transformants were selected on Lysogeny broth (LB) agar plates containing 100μg/ml ampicillin at 37°C. Colony PCR was performed on randomly selected individual colonies using pJET1.2-F (5'-CGACTCACTATAGGGAGAGCGGC-3') and pJET1.2-R (5'-AAGAACATCGATTTTCCATGGCAG-3') vector primers using the previously detailed cycling conditions, except the primer annealing temperature was reduced to 56°C. PCR products were analyzed on a 1% (*w*/*v*) agarose/TAE gel and colonies containing the expected size amplification product were grown overnight in 10ml LB containing 100μg/ml ampicillin at 37°C with shaking at 200rpm. Plasmid DNA was isolated from each clone using Wizard® *Plus* SV Miniprep kit (Promega, Madison, WI, United States) and a total of 72 individual clones were sequenced with pJET1.2-F and pJET1.2-R primers at Eurofins Genomics Germany GmbH.

To assess the geographical association between *D. gallinae* and *Rickettsiella*, we used DNA from a previously published mite collection from 63 sites across Europe (Karp-Tatham et al., 2020). For each collection site, DNA extracted from a single mite was screened by PCR for the presence of *Rickettsiella*. Diagnostic *Rickettsiella* primers Rick-F (5'-GTCGAACGGCAGCACGGTAAAGACT-3') and Rick-R (5'-TCGGTTACCTTTCTTCCCCACCTAA-3') were designed based on *Rickettsiella* specific 16S rRNA regions using alignments in the PhylOPDb database (Jaziri et al., 2014). These primers were designed to amplify a 408bp fragment of the *Rickettsiella* 16S rRNA gene. The diagnostic *Rickettsiella* 16S rRNA PCR primers were checked for specificity by running *in silico* searches against the current RDP 16S rRNA database (Wang et al., 2007). In addition, the specificity of the primers was validated by PCR on DNA isolated from adult female *D. gallinae* mites. Amplification products were analyzed by on a 1% (*w*/*v*) agarose/TAE gel to check for size and sequenced, confirming *Rickettsiella* specific amplification.

For screening European mite DNA, each 25μl PCR reaction contained template DNA (5ng), 0.5U Phusion™ High-Fidelity DNA Polymerase (Thermo Fisher Scientific, Waltham, MA, United States), 1× PCR buffer, 0.2mM of each dNTP and each primer at 0.5μM. Cycling conditions were as follows: 98°C for 30s; 30 cycles of 98°C 10s, 68°C 30s, 72°C 30s, and a final hold of 72°C for 10min. All PCR products were analyzed on a 1% (*w*/*v*) agarose/TAE gel and sequenced in both directions using Rick-seq-F (5'-AACGGCAGCACGGTAAAGAC-3') and Rick-seq-R (5'-AGTGCTTTACAACCCGAAGG-3') primers at Eurofins Genomics Germany GmbH.

16S rRNA sequences were classified with the RDP Classifier 2.13 (training set No. 18; Wang et al., 2007) and sequences with <80% bootstrap support as their genus assignment were removed from the dataset. All remaining sequences were used in blastn searches against the GenBank database to identify their top hit.

### Genome Sequencing and Assembly

For the *Rickettsiella* genome assembly, we used PacBio reads from *D. gallinae* eggs that were generated in a previous study (Burgess et al., 2018). Sequence reads were derived from adult female mites collected at the same commercial laying hen facility as described in section “Mite Collection and Endosymbiont-Enriched DNA Preparation.” The data set contained 7,318,092 reads for a total of 63,984,748,667 bases. Raw reads were mapped against the *D. gallinae* reference genome using Minimap2 v.2.17 (Li, 2018) and unmapped reads were extracted from the resulting BAM files using SAMtools v1.11 (Li et al., 2009). Unmapped reads (814,785 reads for a total of 1,274,422,647 bases) were assembled using the metaFlye assembler v.2.8.2 under default settings using the --pacbio-raw and --meta flags (Kolmogorov et al., 2020). The assembly containing 652 contigs was visualized with Bandage (Wick et al., 2015), which allowed identification of a circular 1.89Mbp *Rickettsiella* genome with 12× coverage.

For massive parallel sequencing (MPS) host-depleted gDNA was extracted from mixed life-stage mites, *D. gallinae* mites collected from a commercial egg laying facility, as described in section “Mite Collection and Endosymbiont-Enriched DNA Preparation.” DNA was fragmented using a Covaris system, size-selected for 200–400bp fragments and used to construct a single strand DNA circle library. The library was amplified using phi29 DNA polymerase by rolling circle amplification to make DNA nanoballs (DNBs) and sequenced on a DNBSEQ-G50 platform as 150bp paired end reads. Library construction and sequencing were performed by BGI Genomics (Shenzhen, China). This sequencing effort resulted in generation of 174,890,018 reads for a total of 26,233,502,700 bases. The reads were used to polish the *Rickettsiella* consensus sequence. Briefly, short-reads were mapped to the *Rickettsiella* genome using BWA-MEM aligner v0.7.17 (Li, 2013) and base calls were corrected using five iterative rounds of polishing with Pilon v1.23 (Walker et al., 2014). The resultant assembly consisted of a single circular chromosome of 1,888,715bp with 3,712× coverage.

### Genome Annotation

The genome was annotated using Prokka v.1.14.6 (Seemann, 2014) and the automated pipeline included coding region prediction by Prodigal (Hyatt et al., 2010) and annotation of non-coding rRNAs using Barrnap and tRNAs using ARAGORN (Laslett and Canback, 2004). As part of the Prokka pipeline, insertion sequences (IS) were annotated using the ISfinder database (Siguier et al., 2006). Candidate pseudogenes were identified based on the length ratios of each predicted *Rickettsiella* DGE protein against their top blastp hit from searches against the NCBI nr protein database. *Rickettsiella* DGE proteins that deviated by +/− 25% compared to their top blastp hit were flagged as potential pseudogenes. Metabolic pathways for amino acids, B vitamins and cofactors were manually inspected using KEGG (Kanehisa and Goto, 2000) and MetaCyc (Caspi et al., 2006) reference pathways. The absence of genes in pathways was verified by tblastn searches against the *Rickettsiella* genome. The *Rickettsiella* DGE genome plot was generated using DNAplotter (Carver et al., 2009). Synteny analysis was performed between the *Rickettsiella* DGE and *Rickettsiella viridis* genome (accession number: AP018005) using MUMmer (Delcher et al., 2002; nucmer --maxgap=1,000 -mumreference -c 100).

### Phylogenetic Analysis

A phylogenetic relationship of *Rickettsiella* isolates was reconstructed using 16S rRNA sequences obtained from *D. gallinae*. To reconstruct the *Rickettsiella* phylogeny, we retrieved additional 16S rRNA sequences from GenBank based on sequence similarity to the *Rickettsiella* DGE 16S sequence and more distantly related bacteria. This dataset included sequences from *Rickettsiella* found in various tick species, insect species, and other arthropods. Using this dataset 16S rRNA sequences were aligned using ClustalW (1,013bp unambiguously aligned sites) and a maximum-likelihood (ML) phylogenetic tree constructed using the Kimura 2-parameter (K2) model with gamma distributed with invariant sites (G+I). The substitution model was selected based on BIC score (Bayesian Information Criterion) and reliability of the tree was tested using bootstrap analysis (1,000 replicates) with bootstrap values indicated on the tree. All phylogenetic analyzes were performed using MEGA version X (Kumar et al., 2018).

## Results and Discussion

### *Rickettsiella* Is Present in *Dermanyssus gallinae* Eggs

**Figure 1:**
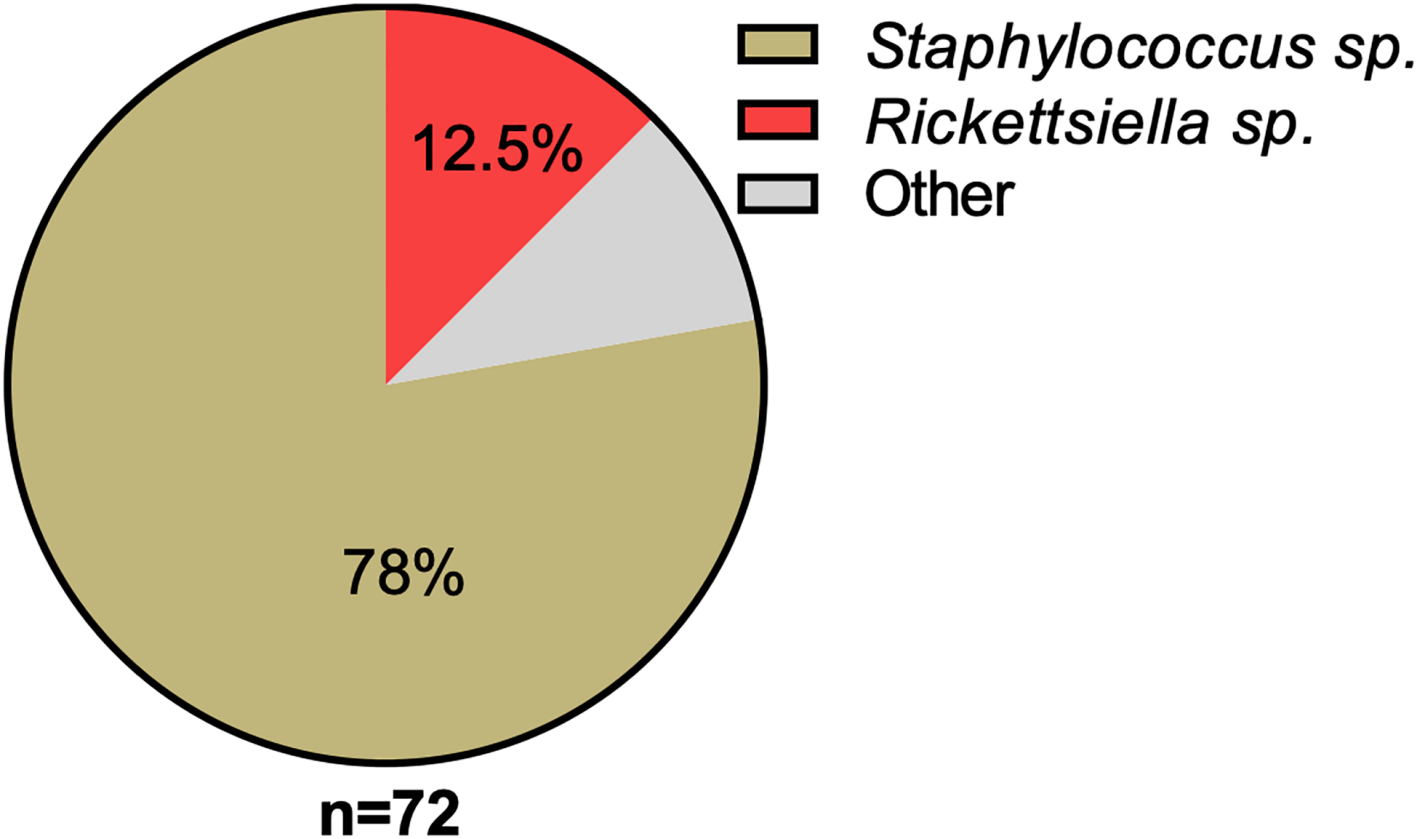
Classification and relative abundance of bacteria associated with *Dermanyssus gallinae* eggs. The presence of bacterial DNA in mite eggs was verified by PCR using universal bacterial 16S rRNA gene primers. We generated a total of 72 16S rRNA reads from DNA isolated from surface-sterilized *D. gallinae* eggs. Amplicons were sequenced (*n*=72) and classified with the RDP Classifier 2.13 (training set No. 18). Sequences with <80% bootstrap support as their genus assignment were removed from the dataset. Classifications are as indicated in the legend, other (gray) represents single hits (*n*=1) to the following genera: *Blautia*, *Clostridium*, *Devosia*, *Paenalcaligenes*, *Salinicoccus*, *Streptococcus*, and *Tsukamurella*.

16S rRNA amplicon sequencing of DNA isolated from a pool of surface-sterilized *D. gallinae* eggs reveals that *Rickettsiella* is detectable in mite eggs (Figure 1). From the 72 16S rRNA reads that were generated the majority of reads were from *Staphylococcus* sp. (56 reads, 78% of total reads), while the remainder were from *Rickettsiella* sp. (nine reads, 12.5% of total reads) and single reads (one read for each) to the following genera: *Blautia*; *Clostridium*; *Devosia*; *Paenalcaligenes*; *Salinicoccus*; *Streptococcus*; and *Tsukamurella* (Figure 1). Previous studies of the *D. gallinae* microbiome have identified *Rickettsiella* in all life-stages, including eggs, from mites collected from four geographically isolated commercial laying hen facilities in Czechia (Hubert et al., 2017). *Rickettsiella* is an obligate intracellular bacterium, therefore, it is not likely to be surface associated but found within cells of the mite egg, this raises the possibility that *Rickettsiella* is maternally inherited in *D. gallinae*.

### *Rickettsiella* Infection Is Widespread in European Populations of *Dermanyssus gallinae*

**Figure 2:**
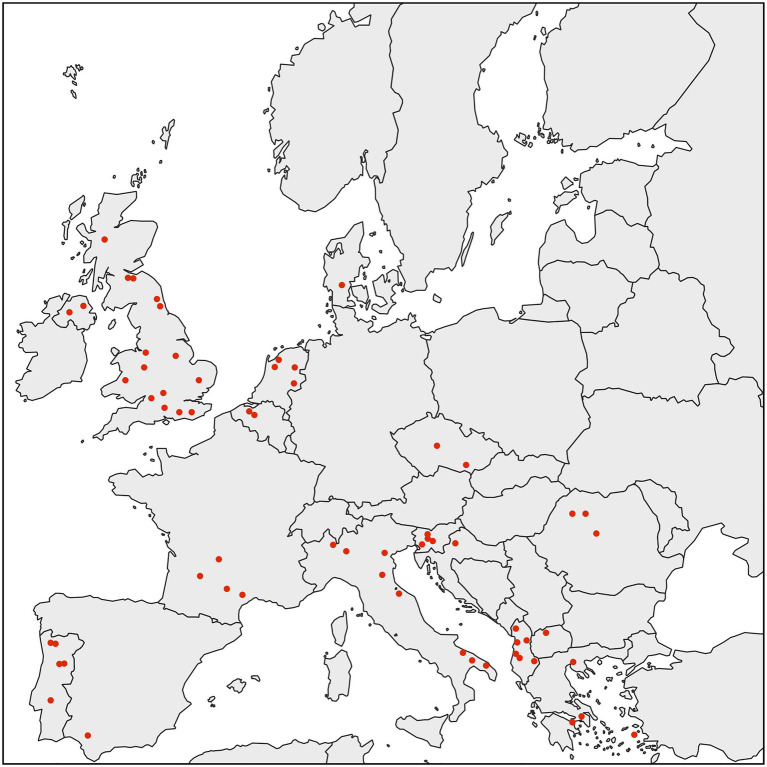
Map showing the distribution of *D. gallinae* populations analyzed in this study. All individual adult female *D. gallinae* mites from each sampling site (63 sites across Europe) were positive for *Rickettsiella* infection (red circle) suggesting that *Rickettsiella* infection may have reached fixation in European *D. gallinae* populations.

We performed an extensive diagnostic PCR screen to test *D. gallinae* from collection sites throughout Europe for the presence of *Rickettsiella*. To do this, we used a previously produced *D. gallinae* DNA archive from mites sourced from commercial laying hen facilities from 63 sites across 15 European countries (Karp-Tatham et al., 2020). For each sample site, total *D. gallinae* DNA from a single adult mite was screened by diagnostic PCR using *Rickettsiella*-specific 16S rRNA primers. All *D. gallinae* DNA samples were *Rickettsiella* positive (*n*=63), indicating that *Rickettsiella* infection is widespread in European *D. gallinae* populations (Figure 2). It is known that other animal- and plant-parasitic arthropods are associated with *Rickettsiella*. For example, non-pathogenic strains of *Rickettsiella* have been reported in the pea aphid *Acyrthosiphon pisum* (Tsuchida et al., 2010, 2014), leafhopper *Orosius albicinctus* (Iasur-Kruh et al., 2013), and ticks *Ixodes woodi* and *Ixodes uriae* (Kurtti et al., 2002; Duron et al., 2016). These strains of *Rickettsiella* are maternally inherited and can reach high frequencies in natural populations (Kurtti et al., 2002; Iasur-Kruh et al., 2013; Tsuchida et al., 2014; Duron et al., 2016). To date, most studies have focused on characterization of *Rickettsiella* population genetics and association with arthropods through sequence analysis of the16S rRNA gene. Therefore, to gain further insight into the biology of *Rickettsiella* from *D. gallinae*, we isolated DNA from mites and completed the *Rickettsiella* DGE genome sequence.

### General Features of the *Rickettsiella* DGE Genome

**Figure 3:**
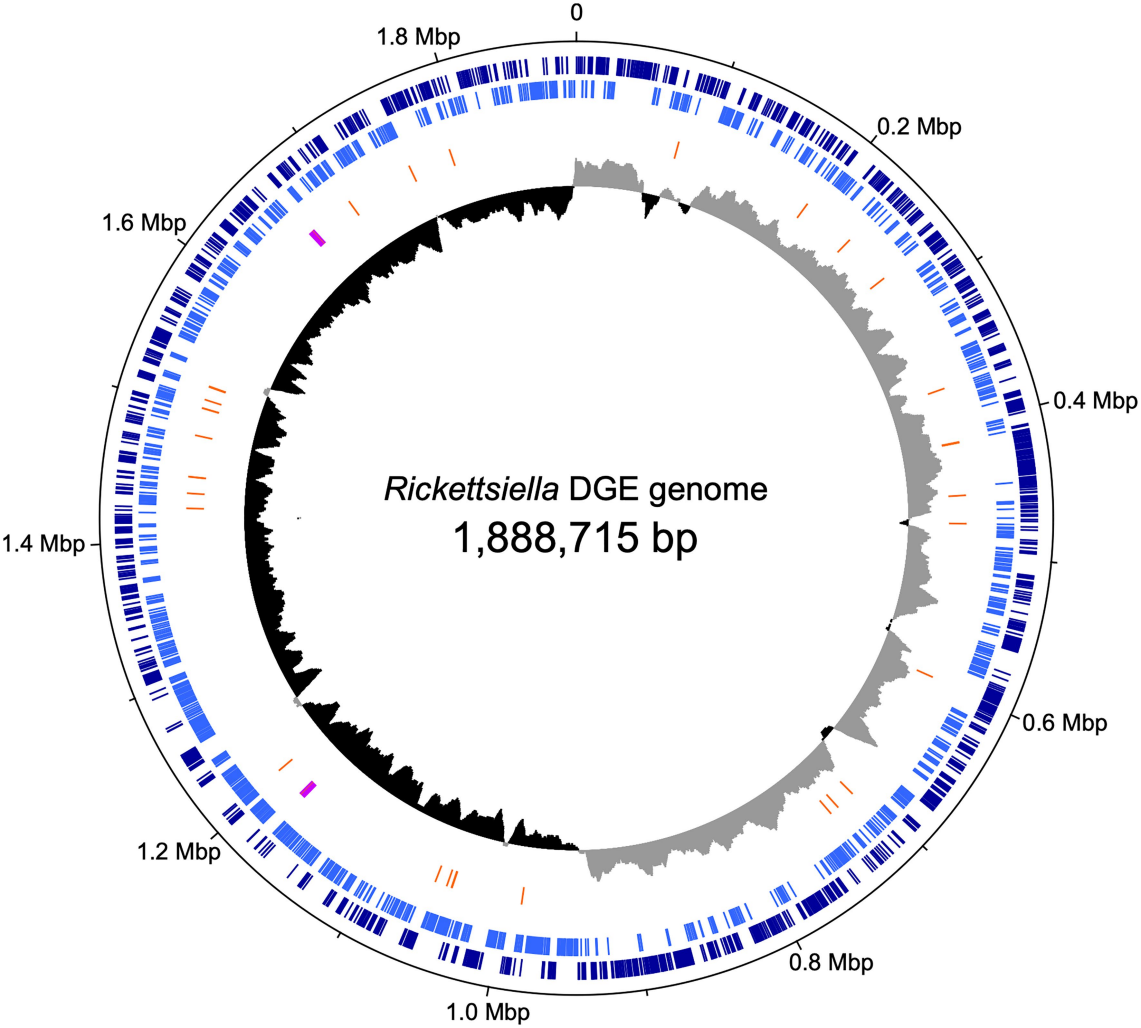
Map of the circular chromosome of *Rickettsiella* DGE. The innermost circle shows GC skew (window size: 10,000bp) with gray and black indicating high (>0) and low (<0) (G−C)/(G+C) values. The second circle shows the positions of tRNA genes (orange) and rRNA genes (purple). The outer circles indicate the positions of protein coding genes on the plus strand (dark blue) and minus strand (light blue).

We used previously-generated PacBio long-read sequence data from *D. gallinae* eggs (Burgess et al., 2018) to assemble the *Rickettsiella* DGE genome. From a total of 64.0Gbp of sequence data, 1.3Gbp of reads did not map to the *D. gallinae* draft genome and were used for metagenome assembly. The metagenome assembly contained 652 contigs and included a circular *Rickettsiella* DGE chromosome of 1.89Mbp. To correct errors associated with long-read sequence data, the *Rickettsiella* DGE assembly was polished using five iterative rounds of Pilon with DNBSEQ™ short-read sequence data from symbiont enriched DNA. This yielded a circular chromosome of 1,888,715bp with 3,712× coverage and a G+C content of 39.6% (Figure 3). Based on Prokka gene prediction and annotation, the *Rickettsiella* DGE genome has 1,973 protein coding open reading frames (ORFs) with an average size of 870bp which covered 91% of the genome (Table 1; Supplementary Table 1). Of these ORFs, 970 were assigned a biological function by Prokka annotation, 585 were annotated by BLAST homology to characterized proteins, while 227 matched hypothetical proteins of unknown function and 191 were unique to *Rickettsiella* DGE. In seven cases, pairs of adjacent genes were annotated with identical names and clearly the ORF was interrupted by a stop codon splitting the gene into two or more parts (these genes are highlighted in Supplementary Table 1). It is likely that these fragmented genes are non-functional and in the early stages of pseudogenization. Other candidate pseudogenes were identified based on their length ratios of each predicted *Rickettsiella* DGE protein against their top blastp hit from searches against the NCBI nr protein database. In summary, out of a total of 1,973 *Rickettsiella* DGE protein coding ORFs searched, only 312 (15.8%) deviate by more than +/− 25% from their top hit and are candidate pseudogenes (Supplementary Table 1). However, it should be noted that the majority of these pseudogene candidates are “hypothetical proteins” of unknown function and, therefore, await experimental validation as genuine loss of function pseudogenes. We detected 41 tRNA genes (which can translate all 61 amino acid codons), six rRNA gene operons and 19 IS elements. *Rickettsiella* DGE contains 19 IS elements evenly distributed around the genome and there are eight copies of IS256 family transposase; four IS481; four ISNCY; and three IS5. Of these IS elements, two IS5 family elements have identical nucleotide sequences (OFBDPGAJ_01174 and OFBDPGAJ_01246) and seven IS256 family elements have identical nucleotide sequences (OFBDPGAJ_00304; OFBDPGAJ_00358; OFBDPGAJ_00392; OFBDPGAJ_00512; OFBDPGAJ_01167; OFBDPGAJ_01364; and OFBDPGAJ_01423). Due to high sequence similarity, it is likely that these IS elements are recently active in the *Rickettsiella* DGE genome.

**Table 1:** General genomic features of *Rickettsiella* DGE and allied Gammaproteobacteria.

	*Rickettsiella* DGE	*R. viridis*	*Coxiella burnetii* RSA 493	*Escherichia coli* K-12
Genome size, Mbp	1.89	1.58	2.00	4.64
G+C (%)	39.6	39.3	42.7	50.8
Protein-coding genes	1,973	1,362	1,798	4,242
Number of COGs^#^	1,322	1,033	1,293	3,812
Coding density (%)	91.0	87.1	77.7	85.8
Average gene size	870	1,010	862	939

^#^COG, cluster of orthologous groups.

### *Rickettsiella* DGE Is Related to Endosymbionts and Endoparasites From the Order *Legionellales*

**Figure 4:**
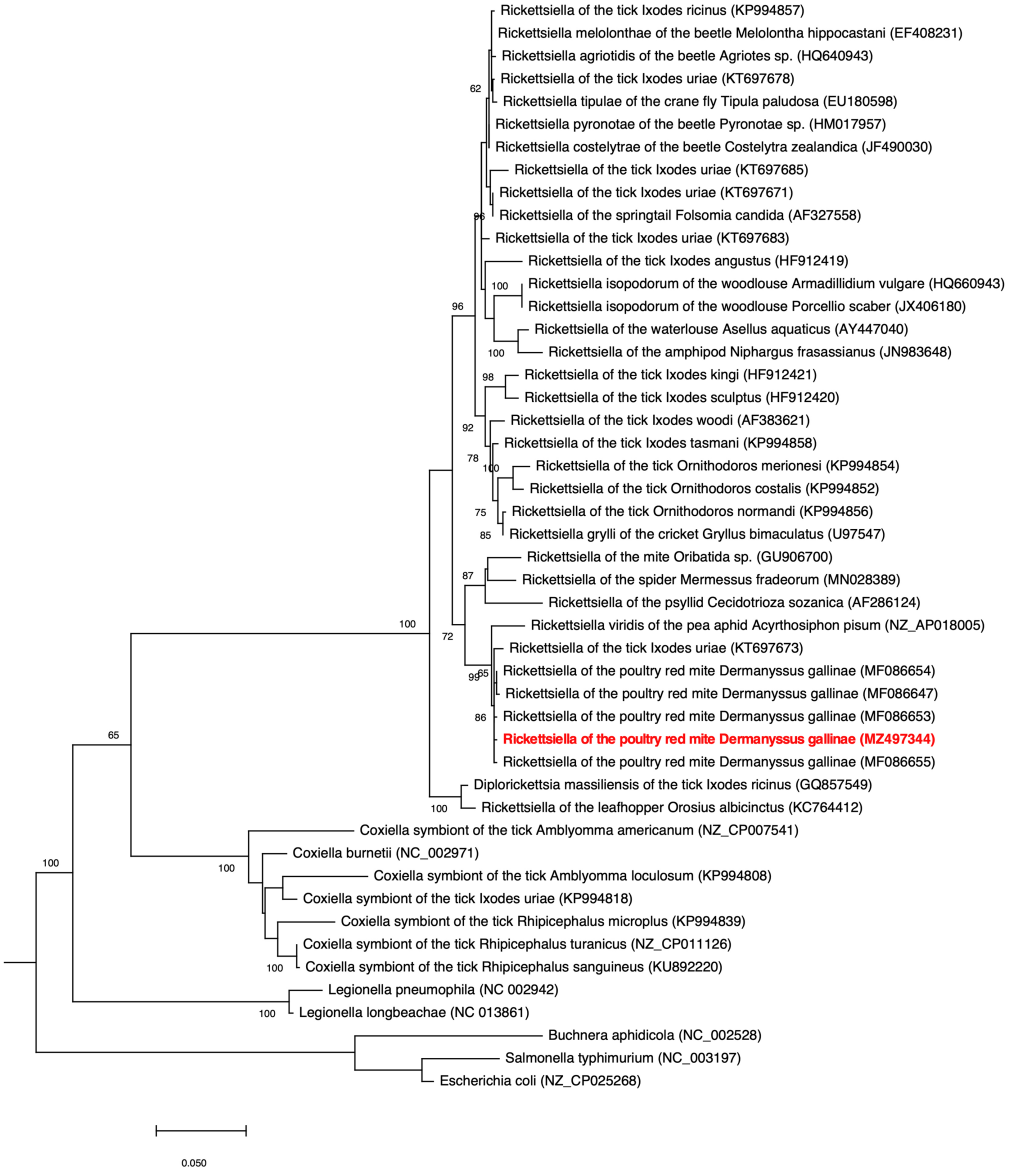
Phylogenetic placement of *Rickettsiella* DGE in the Gammaproteobacteria. The maximum likelihood phylogeny is inferred from 16S rDNA sequences (1,013 unambiguously aligned nucleotide sites). Statistical support is shown at each node from 1,000 bootstrap replicates (bootstrap values>60% are shown). The *Rickettsiella* DGE sequence highlighted in red (MZ497344) was generated in the current study. Accession numbers are indicated in brackets. Scale bar represents 0.02 substitutions per site.

**Figure 5:**
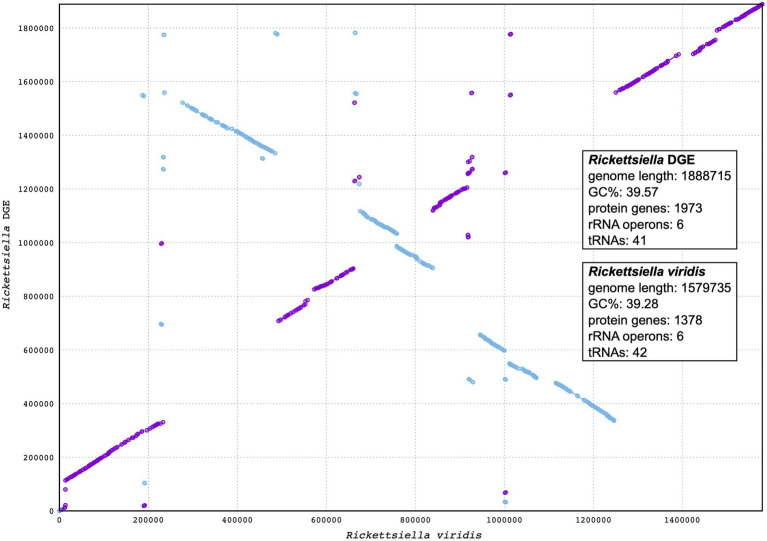
Synteny analysis between *Rickettsiella* DGE and *Rickettsiella viridis* genomes. The *Rickettsiella* DGE genome is represented on the *y* axis and the *R. viridis* genome is represented on the *x* axis. Blue and purple lines represent synteny between the two genomes, with blue lines being inverted in *Rickettsiella* DGE relative to *R. viridis*.

Our phylogenetic analysis, using 16S rRNA gene sequences from representative Gammaproteobacteria, supports the placement *Rickettsiella* DGE within the *Rickettsiella* genus (Figure 4). Members of the *Rickettsiella* genus form a monophyletic group that diverged from *C. burnetii*, the etiologic agent of Q fever, approximately 350million years ago (Cordaux et al., 2007). *Rickettsiella* sp. are found in a wide range of arthropod hosts and are best known as obligate intracellular pathogens (Cordaux et al., 2007; Leclerque and Kleespies, 2008), though, recently, some have been characterized as mutualistic endosymbionts (Tsuchida et al., 2010; Duron et al., 2015). Based on phylogenetic analysis using 16S rRNA sequences, *Rickettsiella* DGE is closely related to *Rickettsiella* that was isolated from *D. gallinae* from commercial egg laying facilities in Czechia (Hubert et al., 2017). Furthermore, all *Rickettsiella* strains from *D. gallinae* are closely related to *Rickettsiella* of the tick *I. uriae* (Duron et al., 2016) and *R. viridis* of the pea aphid, *A. pisum* (Nikoh et al., 2018; Figure 4). In aphids, *R. viridis* infection is associated with production of blue-green pigment molecules that accumulate in the host (Tsuchida et al., 2010) and not associated with negative impacts on host fitness (Tsuchida et al., 2010). As *Rickettsiella* present in *D. gallinae* is closely related to strains found in other species it may indicate horizontal transfer of *Rickettsiella* across arthropod species (Figure 4). Whole genome alignments reveal shared synteny between *Rickettsiella* DGE and *R. viridis*, with evidence of genomic rearrangements including inversions, translocations, and insertions (Figure 5).

*Rickettsiella* DGE is related to other nutritional endosymbionts of blood-feeding arthropods in the order Legionellales. Within the order Legionellales, the CLEs of ticks form a monophyletic group most closely related to the human pathogen *C. burnetii* (Figure 4). In blood-feeding ticks, CLEs are required for the synthesis and supplementation of B vitamins that are lacking in the host’s blood meal and are essential for tick survival (Guizzo et al., 2017). In addition, and again within the order Legionellales, the blood-feeding louse *Polyplax serrata* is associated with a vertically transmitted, host restricted, nutritional endosymbiont from the genus *Legionella* (Říhová et al., 2017; Figure 4). In *P. serrata*, these endosymbionts synthesize and provision B vitamins to their obligate blood-feeding host (Říhová et al., 2017). In summary, endosymbiotic bacteria from the order Legionellales are widely associated with blood feeding arthropods.

### Genomic Reduction in *Rickettsiella* DGE: An Ongoing Process?

Genome reduction is widespread in maternally-inherited bacterial endosymbionts and is associated with loss of genes that are functionally redundant with the host, resulting in compact endosymbiont genomes containing a subset of genes relative to their free-living ancestor (McCutcheon and Moran, 2012). The genome of *Rickettsiella* DGE (1.89Mbp) and *R. viridis* (1.6Mbp) are both moderately reduced in comparison to *C. burnetii* (2.03Mbp; Table 1). Although, it should be noted that *C. burnetii* is already host-adapted as an obligate intracellular parasite and, as such, compared to free-living bacteria it has a degenerate genome (Seshadri et al., 2003). Again, relative to *C. burnetii*, CLEs of blood-feeding ticks have reduced genomes, retaining functionally non-redundant genes that are essential for the symbiosis. Recent genome sequencing studies unveiled that, in comparison to *C. burnetii* (genome size 2.03Mbp), CLEs from ticks exhibit genome reduction, with genomes ranging from 0.66Mbp for *Coxiella* sp. strain CLEAA (CLE of *A. americanum*; Smith et al., 2015) to 1.73Mbp for *Coxiella* sp. strain CRt (CLE of *Rhipicephalus turanicus*; Gottlieb et al., 2015). Presumably the range of genome size among CLEs of blood-feeding ticks reflects an ongoing dynamic process of reductive genome evolution. Metabolic reconstruction of these reduced genomes reveals intact B vitamin biosynthesis pathways, required for biosynthesis and provision of these essential nutrients to the host tick (Gottlieb et al., 2015; Smith et al., 2015).

Perhaps the most striking example of genome reduction, in the transition from a pathogen to a nutritional mutualist, is the loss of virulence associated secretion systems. In the pathogens *C. burnetii* and *Legionella pneumophila* the type IV Dot/Icm secretion system (T4SS) functions to export a suite of virulence factors that modulate host physiology and are essential for establishment and maintenance of infection (Seshadri et al., 2003; Chien et al., 2004; Gomez-Valero et al., 2019). Intriguingly, the massively reduced genomes of *Coxiella* from the lone star tick *A. americanum* (CLEAA) and *Legionella polyplacis* from the blood-feeding louse *P. serrata* do not encode a Dot/Icm type IVB secretion system and presumably this secretion apparatus is not required in these nutritional mutualists (Smith et al., 2015; Říhová et al., 2017). In contrast, components of the Dot/Icm type IVB secretion system are retained in *Rickettsiella* DGE and are also present in the closely related genome of *R. viridis*, although, the sequences of core components are highly divergent when compared with *L. pneumophila* orthologs (Supplementary Table 2). It therefore remains to be determined if the Dot/Icm type IVB secretion system is functional in *Rickettsiella* DGE and the role it plays in cellular interactions with the host.

### Metabolic Capacity of *Rickettsiella* DGE: A Putative Nutritional Mutualist

**Figure 6:**
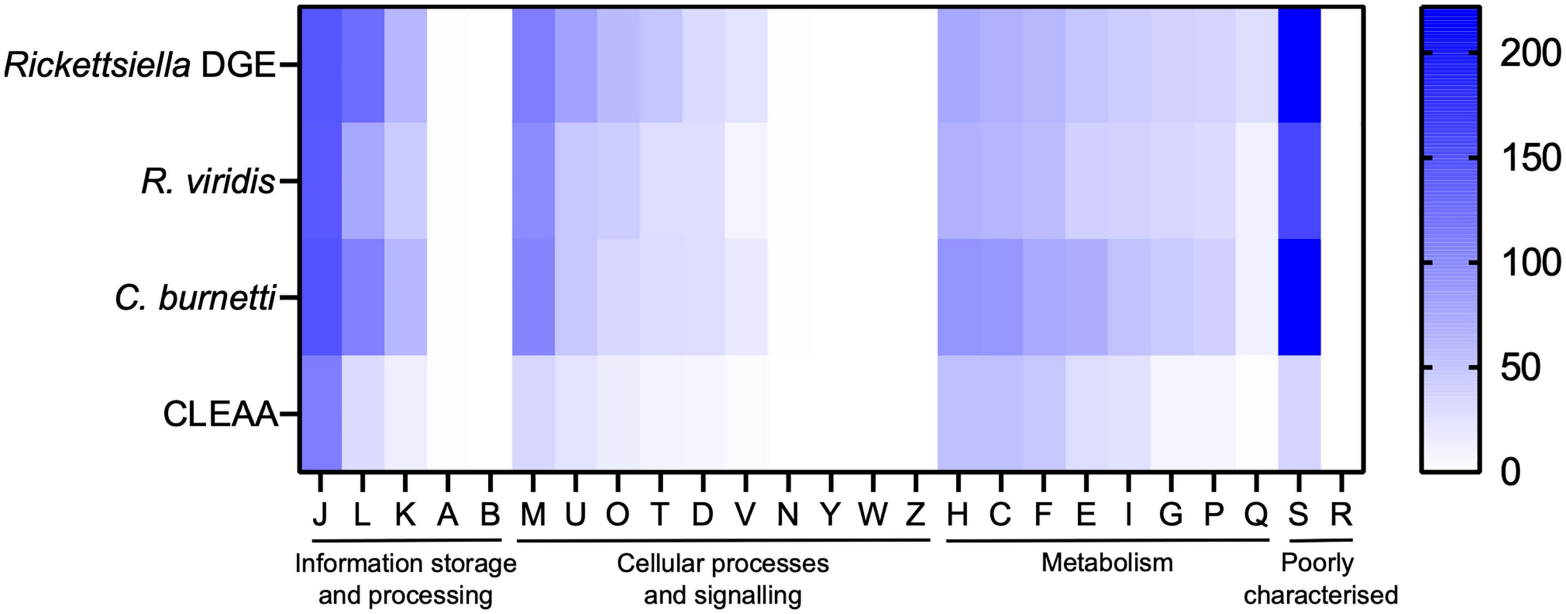
Heatmap comparison of cluster of orthologous groups (COG) frequency in *Rickettsiella* DGE and related bacteria. Abbreviations for functional categories are as follows: J, translation, ribosomal structure, and biogenesis; L, replication, recombination, and repair; K, transcription; A, RNA processing and modification; B, chromatin structure and dynamics; M, cell wall/membrane/envelope biogenesis; U, intracellular trafficking, secretion, and vesicular transport; T, signal transduction mechanisms; O, posttranslational modification, protein turnover, chaperones; D, cell cycle control, cell division, chromosome partitioning; V, defense mechanisms; N, cell motility; Y, nuclear structure; W, extracellular structures; Z, cytoskeleton; H, coenzyme transport and metabolism; C, energy production and conversion; F, nucleotide transport and metabolism; E, amino acid transport and metabolism; I, lipid transport and metabolism; G, carbohydrate transport and metabolism; P, inorganic ion transport and metabolism; Q, secondary metabolites biosynthesis, transport, and catabolism; S, function unknown; and R, general function prediction only. Scale bar (0, white; 200, blue) indicates number of COGs in each category.

The *Rickettsiella* DGE genome, as with the related intracellular facultative symbiont *R. viridis*, retains genes for basic cellular processes including translation, replication, cell wall biosynthesis, and energy production (Figure 6). In Supplementary Table 3, we provide a more detailed comparative gene content analysis between *Rickettsiella* DGE and genomes of *R. viridis* and *C. burnetii* using the pathway/gene list published by Moran et al. (2008) and Bennett and Moran (2013).

**Figure 7:**
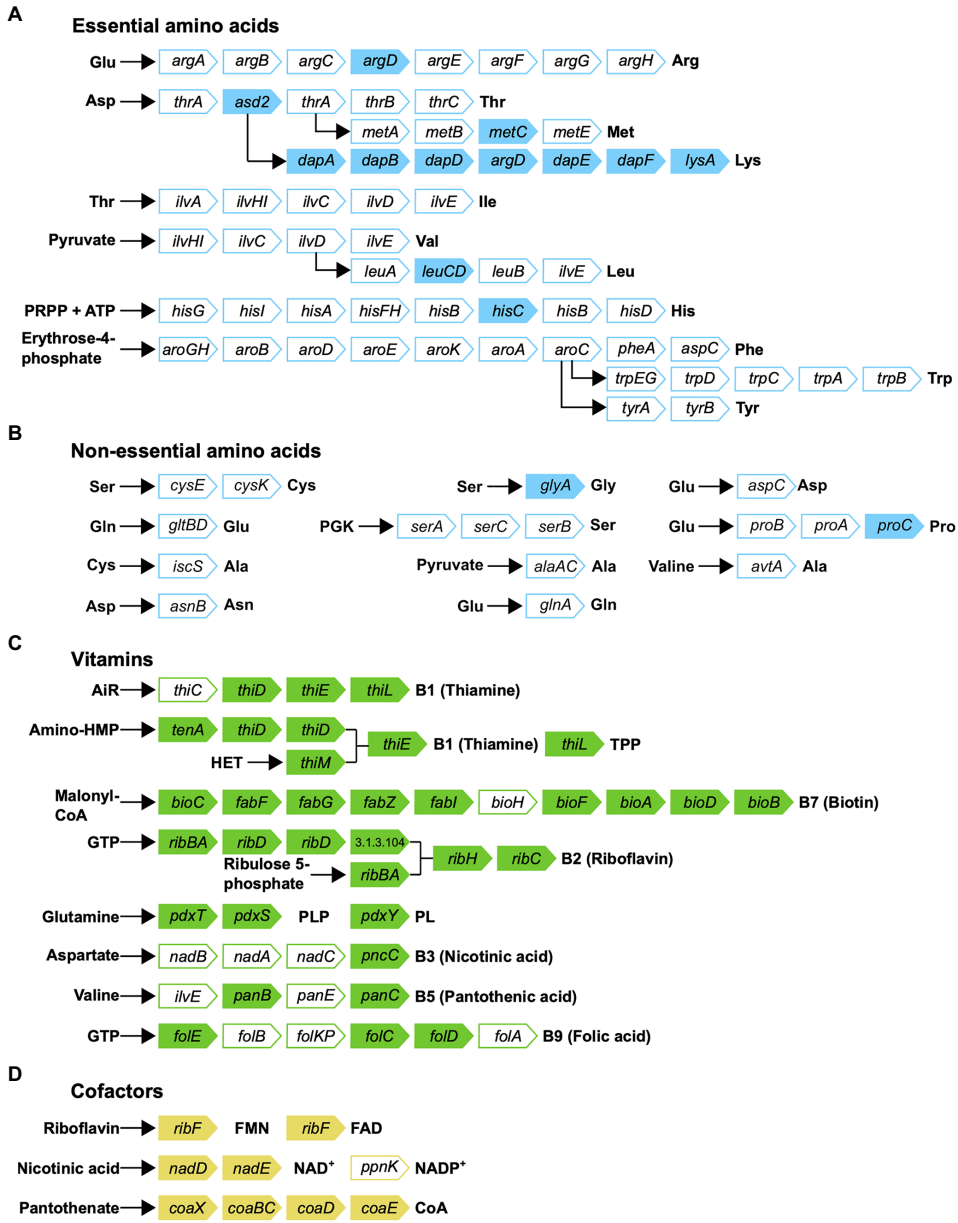
Biosynthetic pathways for synthesis of **(A)** essential amino acids; **(B)** non-essential amino acids; **(C)** vitamins; and **(D)** cofactors in *Rickettsiella* DGE. Gene names are indicated in arrowed rectangles; colored arrows show genes present in *Rickettsiella* DGE; missing genes are shown in white arrows.

Metabolic reconstruction of amino acid biosynthesis pathways revealed that *Rickettsiella* DGE is unable to synthesize protein amino acids and, therefore, amino acids are likely provisioned by the host (Figure 7). The biosynthesis pathway for the essential amino acid lysine is mostly complete (8/9 required genes present), although, precursor aspartic acid in not synthesized by *Rickettsiella* DGE and the bifunctional aspartokinase/homoserine dehydrogenase 1 (encoded by *thrA*) is missing, again suggesting this pathway is non-functional. Given that *D. gallinae* feeds on blood and is able to digest hemoglobin and other blood proteins to release free amino acids (Price et al., 2019), it likely has an excess of essential and non-essential amino acids that meet its own nitrogen requirements and those of *Rickettsiella* DGE. Indeed, in other nutritional endosymbionts of obligate blood feeding arthropods, amino acid biosynthesis pathways are absent and it is likely the host supplies amino acids to the endosymbiont (Chien et al., 2004; Smith et al., 2015; Duron et al., 2018).

Obligate blood feeding arthropods such as the human body louse (*Pediculus humanus*; Kirkness et al., 2010), African soft tick (*O. moubata*; Duron et al., 2018) and the Lone star tick (*A. americanum*; Smith et al., 2015) depend on nutritional endosymbionts to synthesize and provide B vitamins that are available in trace amounts in mammalian blood (reviewed in Husnik, 2018). Thus, to determine whether *Rickettsiella* DGE is able to synthesize B vitamins, we surveyed its genome for B vitamin biosynthesis genes. The *Rickettsiella* DGE genome has conserved genes involved in the biosynthesis of seven B vitamins, including complete biosynthetic pathways for thiamine (vitamin B1) *via* the salvage pathway, riboflavin (vitamin B2), pyridoxine (vitamin B6) and the cofactors FAD, and CoA (Figure 7). The biosynthesis pathway for biotin (vitamin B7) is largely complete (9/10 genes present), although, it is missing *bioH*, which is required for pimeloyl-CoA synthesis. The annotated biotin biosynthesis pathway is based on that of the model organism *E. coli*, where *bioC* and *bioH* are required for synthesis of the intermediate pimeloyl-CoA. However, unlike the representative “*bioC*/*bioH*” pathway of *E. coli* many *bioC*-containing microorganisms lack *bioH* homologs, raising the possibility of non-homologous gene replacement in some bacteria (Shapiro et al., 2012). To date, there are five documented cases of *bioH* gene replacement, which includes *bioK* of *Synechococcus* (Shapiro et al., 2012), *bioG* of *Haemophilus influenzae* (Shapiro et al., 2012), *bioJ* of *Francisella* sp. (Feng et al., 2014), *bioV* of *Helicobacter* sp. (Bi et al., 2016), and *bioZ* of *Agrobacterium tumefaciens* (Hu and Cronan, 2020). Further tblastn searches against the *Rickettsiella* DGE genome using *bioH* and the non-homologous gene replacements *bioK*, *bioG*, *bioJ*, and *bioV* did not identify gene products that can fill the *bioH* gap. However, a gene encoding ketoacyl-ACP synthase (KAS) III from *Rickettsiella* DGE (gene locus OFBDPGAJ_01014) has similarity to *bioZ* of *A. tumefaciens* (53.8% amino acid similarity) and is therefore a candidate to replace *bioH*. Alignments between *A. tumefaciens* KAS III (*bioZ*) and orthologs from *Rickettsiella* DGE as well as other *Rickettsiella* sp. are shown in Supplementary Figure 1. Given the retention of a long biotin biosynthesis pathway in *Rickettsiella* DGE (9/10 genes present) and the propensity for the missing *bioH* gene to be replaced in other bacteria, we predict that the biotin biosynthesis pathway is functional in *Rickettsiella* DGE. In contrast, the other B vitamin biosynthesis pathways for nicotinic acid (vitamin B3), pantothenic acid (vitamin B5), and folic acid (vitamin B9) are more fragmented and it is not clear if these pathways are functional.

In other nutritional host/endosymbiont interactions it has been shown that some fragmented metabolic pathways of nutritional endosymbionts are functional with gene products supplemented from multiple species including the host and/or symbiont partners. This complex arrangement results in metabolic mosaics for the synthesis of essential nutrients (McCutcheon et al., 2009; Husnik et al., 2013). By utilizing the *D. gallinae* genome (Burgess et al., 2018), we investigated whether host gene products are capable of filling missing steps in *Rickettsiella* DGE B vitamin biosynthesis pathways. In general, animals cannot synthesize B vitamins *de novo*, therefore, we explored the possibility that *D. gallinae* has acquired genes through horizontal gene transfer (HGT) that allows these fragmented pathways to function. To screen for potential HGT events, we used *E. coli* proteins from each of the missing steps in *Rickettsiella* DGE B vitamin biosynthesis as “query” proteins in blastp searches against predicted proteins from the *D. gallinae* genome (Burgess et al., 2018). These searches did not identify candidate genes from *D. gallinae* and it is therefore unlikely that *D. gallinae* contributes to B vitamin biosynthesis by completing these missing steps. Another possibility is that fragmented B vitamin pathways in *Rickettsiella* DGE are completed by gene products from other endosymbionts of the mite. A previous microbiome analysis of *D. gallinae* identified several additional endosymbionts (including *Bartonella*, *Cardinium*, and *Wolbachia*) that are prevalent in mite populations (Hubert et al., 2017). However, the biosynthetic capability of these *D. gallinae* endosymbionts is currently unknown (Hubert et al., 2017). Thus, future work will analyze B vitamin biosynthesis in the context of the *D. gallinae* metagenome.

In addition to blood feeding ticks and mites, many insects are specialist blood feeders (reviewed in Husnik, 2018). To utilize their blood diet, obligate blood feeding insects also associate with mutualistic endosymbionts that are important for provision of B vitamins to the host (Akman et al., 2002; Kirkness et al., 2010; Nikoh et al., 2014). While endosymbiotic partners differ, there are many commonalities between endosymbiotic partners from blood-feeding ticks, mites, and insects (Husnik, 2018). For example, the tsetse fly (*Glossina morsitans*) is critically reliant on its obligate endosymbiont *Wigglesworthia glossinidia*. Elimination of the symbiont using antibiotic treatment results in reproductive failure of the tsetse host (Rio et al., 2016). Critically, it has been shown that reproduction can by partially restored in these flies by dietary supplementation with B vitamins, suggesting that the endosymbiont may provision these nutrients (Rio et al., 2016). Furthermore, in support of its role as a nutritional mutualist, the genome of *Wigglesworthia* reveals that the small 700Kbp endosymbiont genome retains the capability to synthesise B vitamins (Akman et al., 2002; Rio et al., 2012). In comparison to *Rickettsiella* DGE, where we observe several incomplete B vitamin biosynthesis pathways (Figure 7), *Wigglesworthia* has complete pathways for the synthesis of biotin (vitamin B7), thiamine (vitamin B1), riboflavin (vitamin B2), pantothenic acid (vitamin B5), and pyridoxine (vitamin B6; Akman et al., 2002; Rio et al., 2012). Although speculative, this may reflect differing requirements of the host for B vitamin supplementation across these host/endosymbiont systems. Indeed, genome analysis of endosymbionts from other blood feeding arthropods reveals differing levels of completeness of retained B vitamin biosynthesis pathways (Kirkness et al., 2010; Nikoh et al., 2014; Smith et al., 2015; Duron et al., 2018). A recent analysis of endosymbiont genomes from obligate blood feeding arthropods reveals that all genomes analyzed retain “core” biosynthesis pathways for biotin (vitamin B7) and to a lesser degree folic acid (vitamin B9) and riboflavin (vitamin B2; Duron and Gottlieb, 2020). In the analysis by Duron and Gottlieb (2020), other B vitamin pathways were more fragmented and pathway functionality may reflect the lifestyle of the host and its nutritional requirement for B vitamin supplementation. In both *Rickettsiella* DGE and other endosymbionts from blood feeding arthropods further investigation is needed to understand if and how these fragmented B vitamin biosynthetic pathways are functional as well as the exact B vitamin requirements of each host.

Currently, in the *D. gallinae* – *Rickettsiella* DGE endosymbiotic system the tissue location of *Rickettsiella* DGE is unknown, as is the identity of host genes required for the maintenance of the association. Again, this is something that has been extensively investigated in the tsetse/*Wigglesworthia* interaction (Bing et al., 2017). In teste flies, *Wigglesworthia* is located in host bacteriocyte cells that collectively form the bacteriome organ in the anterior midgut (Rio et al., 2012). Dual analysis of the host/endosymbiont transcriptome identified host factors that contribute to the maintenance of the symbiosis and a multivitamin transporter potentially involved in nutrient provision to the host (Bing et al., 2017). In support of its role as a nutritional mutualist, genes involved in biosynthesis of B vitamins and co-factors were highly expressed by the endosymbiont (Bing et al., 2017). Thus, the key priorities for future research are to determine the molecular processes underpinning maintenance of *Rickettsiella* DGE in host cells and the genetic and metabolic mechanisms by which nutrient flux between host and endosymbiont is regulated.

## Data Availability Statement

DNBseq reads were deposited to the Sequence Read Archive (SRA), under NCBI BioProject PRJNA743410. The genome sequence of *Rickettsiella* DGE has been deposited at GenBank with the accession number CP079094. *Rickettsiella* DGE 16S rRNA generated in this study is available from GenBank under the following accession numbers: MZ497336–MZ497344.

## Author Contributions

DP, AN, and SB conceived the study and analyzed the data. DP, KB, DB, EK-T, FN, SB, and AN designed the research. DP and EK-T performed the research. DP wrote the paper with contributions from all authors. All authors contributed to the article and approved the submitted version.

## Conflict of Interest

The authors declare that the research was conducted in the absence of any commercial or financial relationships that could be construed as a potential conflict of interest.

## Publisher’s Note

All claims expressed in this article are solely those of the authors and do not necessarily represent those of their affiliated organizations, or those of the publisher, the editors and the reviewers. Any product that may be evaluated in this article, or claim that may be made by its manufacturer, is not guaranteed or endorsed by the publisher.
