# Supplementary material for: A Rickettsiella Endosymbiont Is a Potential Source of Essential B-Vitamins for the Poultry Red Mite, Dermanyssus gallinae
Source: Front Microbiol. 2021 Sep 3;12:695346. doi: 10.3389/fmicb.2021.695346 (PMC8446615; doi:10.3389/fmicb.2021.695346)
Supplement: Supplementary file 1 [file Data_Sheet_1.DOCX]

Agrobacterium_tumefaciens[6KUE] MQTRSSRMAGFGHAVPARCVDNAEIEA-SLGLEAGWIERRTGIRSRYWAEAGDTLSGLAE 59

Rickettsiella_DGE[OFBDPGAJ_01014] --MKYSRIAGTGAYLPKNSVSNADLIKRGIDTTDEWIVSRTGIRARHLADASDTTSTMAA 58

Rickettsiella_viridis[WP_126322709] --MKYSRIAGTGAYLPKNSVSNADLIKRGIDTTDEWIVSRTGIRARHLADASDTTSTMAT 58

Diplorickettsia_massiliensis[WP_202798011] ---MFSKIEGTGSYLPKNILTNADLMAKGIETSHEWIVTRTGIHQRHVVNAEETTATMAV 57

Rickettsiella_isopodorum[WP_071662275] --MKYSRIAGTGSYLPEKILTNAELEK-KVATTNEWIVERTGIRERRIIGPNDSTASMAA 57

Rickettsiella_grylli[WP_071660953] --MKYSRIAGTGSYLPEKILTNAELEK-KVATTNEWIVARTGIRERRIIGPDDSTASMAV 57

*:: * * :* . : **:: : ** ****: * :: : :*

Agrobacterium_tumefaciens[6KUE] RAGRMALEDAKINADDIALTLLATSTPDHLLPPSAPLLAHRLGLTRSGAIDLAGACSGFL 119

Rickettsiella_DGE[OFBDPGAJ_01014] AAAKKALEAAGCAAKDIQLIIVATSTPDKYFPSTACLVQSALAVDGCPAFDVVAACAGFN 118

Rickettsiella_viridis[WP_126322709] AAAKKALEAAGCSAKEIQLIIVATSTPDKYFPSTACLVQSALGVDGCPAFDVVAACAGFN 118

Diplorickettsia_massiliensis[WP_202798011] AAAQQALYAAQCTAQEIELIIVATSTPDRFFPSTACLVQATLGVDGCPAFDVVAACSGFN 117

Rickettsiella_isopodorum[WP_071662275] AAAKQALLAAEMVAKDIQLIIVATSTPDKFFPSTACLVQAELGEDGCPSFDIVAACAGFN 117

Rickettsiella_grylli[WP_071660953] AAAQHALLAASISPKNIQLIIVATSTPDKFFPSTACLVQAGLGYDGCPAFDIVAACAGFN 117

*.: ** * .:* * ::******: :* :* *: *. . ::*:..**:**

Agrobacterium_tumefaciens[6KUE] YALTLADGFVRTYG-RAVLVAAANILSRRINPAERASAVLFADAAGAVVLTPCPEVKRGV 178

Rickettsiella_DGE[OFBDPGAJ_01014] YALSVADQFIRNGQINSALVIGSETMSSIINWEDRSTCILFGDGAGAVVLQASD--TPGI 176

Rickettsiella_viridis[WP_126322709] YALSVADQFIRNGQINSALVIGSETMSSIINWEDRSTCILFGDGAGAVVLQASD--TPGI 176

Diplorickettsia_massiliensis[WP_202798011] YALSVADQFIRNGQVKTALVIGSETMSSIVNWQDRSTCILFGDGAGAVVLKAAS--EPGI 175

Rickettsiella_isopodorum[WP_071662275] YALSVADQFIRNGVVETALVIGSESMSNIINWEDRSTCILFGDGAGAVVLQAAK--EPGI 175

Rickettsiella_grylli[WP_071660953] YALSVADKFIRSGTVETALVIGSESMSTIINWEDRSTCVLFGDGAGAVVLQAAK--EPGI 175

***::** *:*. .:.** .:: :* :* :*::.:**.*.****** . *:

Agrobacterium_tumefaciens[6KUE] LSADLVADGSGYDLIQIAAGGSSQPFSAGMIAEDALMTMRDGREVFSRAVALMTNTSQRV 238

Rickettsiella_DGE[OFBDPGAJ_01014] ISTHLHAAGSYKDLLFLGTG---------LREE-KAHLVMQGNKVFKIAVSKLSEILDET 226

Rickettsiella_viridis[WP_126322709] ISTHLHAAGSYKDLLFLGTG---------LREE-KAHLVMQGNPVFRIAVKKLSEILDET 226

Diplorickettsia_massiliensis[WP_202798011] ISTHLKAAGSHKDLLYLGTG---------LRDE-EAYLVMEGREIFKLAVKNLSSILDET 225

Rickettsiella_isopodorum[WP_071662275] ISAHLHAAGSYKDLLSLGTG---------LKKGDVPHLKMQGSEVFKIAVNKLSAVLEET 226

Rickettsiella_grylli[WP_071660953] ISAHLHAAGSYKDLLSLDTG---------LKKGDLPQLKMQGSEVFKIAVNKLSSVLEET 226

:*:.* * ** **: : :* : :* :* ** :: :..

Agrobacterium_tumefaciens[6KUE] LHEAELTAADISRFVPHQANARMSDAVCGNLGIEREKTVRTIGSFGNSSAATIPLSLSIT 298

Rickettsiella_DGE[OFBDPGAJ_01014] LQANNIQASTIDWLIPHQANLRIITAMADKLKLSMDKVVITVDQHGNTSAASVPLALDTA 286

Rickettsiella_viridis[WP_126322709] LQANGIQASAIDWLIPHQANLRIITAMADKLKLSMDKVVITVDQHGNTSAASVPLALDTA 286

Diplorickettsia_massiliensis[WP_202798011] LQANGIKPEEIDWLIPHQANLRIIKAMAQKLAMPMEKVIVTVDQHGNTSAASIPLALDQS 285

Rickettsiella_isopodorum[WP_071662275] LTANKINKKDIDWLIPHQANLRIIQAMANKLKMTLQQVVVTLDQHGNTSAASIPLALDVA 286

Rickettsiella_grylli[WP_071660953] LASNKIDKEDIDWFIPHQANLRIIKAMANKLKMTLDQVVITLDQQGNTSAASIPLALDSA 286

* : *. ::***** *: *:. :* : ::.: *:.. **:***::**:*. :

Agrobacterium_tumefaciens[6KUE] NAERPLAGGETLLLTAAGAGMTGGAVVYRV 328

Rickettsiella_DGE[OFBDPGAJ_01014] IRDGRIQRGQLLLMESFGAGLAWGSALVRY 316

Rickettsiella_viridis[WP_126322709] VREGRIQRGQLLLMESFGAGLAWGSALVRF 316

Diplorickettsia_massiliensis[WP_202798011] VRDGRLQRGQLFLMESFGAGLAWGSALVRY 315

Rickettsiella_isopodorum[WP_071662275] VRDGRVQRGQLLLMESFGAGLTWGSALVRY 316

Rickettsiella_grylli[WP_071660953] VRDGRIQRGQLLLMESFGAGLAWGSALVRY 316

: : *: :*: : ***:: *:.: *

**Supplementary Figure 1.** Clustal Omega alignment of ketoacyl-ACP synthase (KAS) III from *Agrobacterium tumefaciens* (6KUE) and related sequences from: *Rickettsiella* DGE (gene locus OFBDPGAJ_01014); *Rickettsiella viridis* (WP_126322709); *Diplorickettsia massiliensis* (WP_202798011); *Rickettsiella isopodorum* (WP_071662275) and *Rickettsiella grylli* (WP_071660953). Asterisks indicate identical residues in all sequences; colons indicate conservative amino acid substitutions; dots indicate semi-conservative amino acid substitutions.
